# Alterations in bone fracture healing associated with TNFRSF signaling pathways

**DOI:** 10.3389/fphar.2022.905535

**Published:** 2022-10-17

**Authors:** Yanzhao Dong, Haiying Zhou, Ahmad Alhaskawi, Zewei Wang, Jingtian Lai, Sohaib Hasan Abdullah Ezzi, Vishnu Goutham Kota, Mohamed Hasan Abdulla Hasan Abdulla, Zhenyu Sun, Hui Lu

**Affiliations:** ^1^ Department of Orthopedics, B Department of Rehabilitation Medicine, the First Affiliated Hospital, Zhejiang University, Hangzhou, China; ^2^ Zhejiang University School of Medicine, Hangzhou, China; ^3^ Alibaba-Zhejiang University Joint Research Center of Future Digital Healthcare, Zhejiang University, Hangzhou, China

**Keywords:** fracture, TNFR1, TNFR2, RANK/RANKL/OPG, healing

## Abstract

Bone fracture healing is a complex process involving various signaling pathways. It remains an unsolved issue the fast and optimal management of complex or multiple fractures in the field of orthopedics and rehabilitation. Bone fracture healing is largely a four-stage process, including initial hematoma formation, intramembrane ossification, chondrogenesis, and endochondral ossification followed by further bone remodeling. Many studies have reported the involvement of immune cells and cytokines in fracture healing. On the other hand, the Tumor Necrosis Factor (TNF) family and TNF receptor superfamily (TNFRSF) play a pivotal role in many physiological processes. The functions of the TNF family and TNFRSF in immune processes, tissue homeostasis, and cell differentiation have been extensively studied by many groups, and treatments targeting specific TNFRSF members are in progress. In terms of bone fracture management, it has been discovered that several members of TNFRSF have very distinct functions in different stages of fracture healing, including TNFR1, TNFR2, and receptor activator of nuclear factor kappa-B (RANK) pathways. More specifically, TNFR1 is associated with osteoclastogenesis and TNFR2 is associated with osteogenic differentiation, while RANK is in association with bone remodeling. In this review, we will discuss and summarize the involvement of members of TNFRSF including TNFR1, TNFR2, and Receptor activator of nuclear factor kappa-B (RANK) pathways in different stages of fracture healing and bone remodeling and the current treatment trend involving TNFRSF agonists and antagonists.

## 1 Background

General background of TNFRSF signaling pathways and clinical application of TNFRSF agonists and antagonists, an overview of the bone regeneration process following bone fracture, and the future perspectives of application of TNFRSF agonists and antagonists in the treatment of bone fracture. TNF was first identified as early as 4 decades ago as a product of lymphocytes that exerts lytic effects against certain cell types, tumor cells in particular. Later in 1975, Carswell et al. chemically and genetically described this cytokine as “tumor necrotizing factors”, which was later called tumor necrosis factor (Granger et al., 1969; Carswell et al., 1975). Using large-scale sequencing, a series of similar proteins were discovered, and collectively they were categorized as TNF superfamily (TNFSF), their receptors, therefore, are categorized as TNF receptor superfamily (TNFRSF) (Idriss and Naismith, 2000).

### 1.1 TNFRSF signaling pathways

**FIGURE 1:**
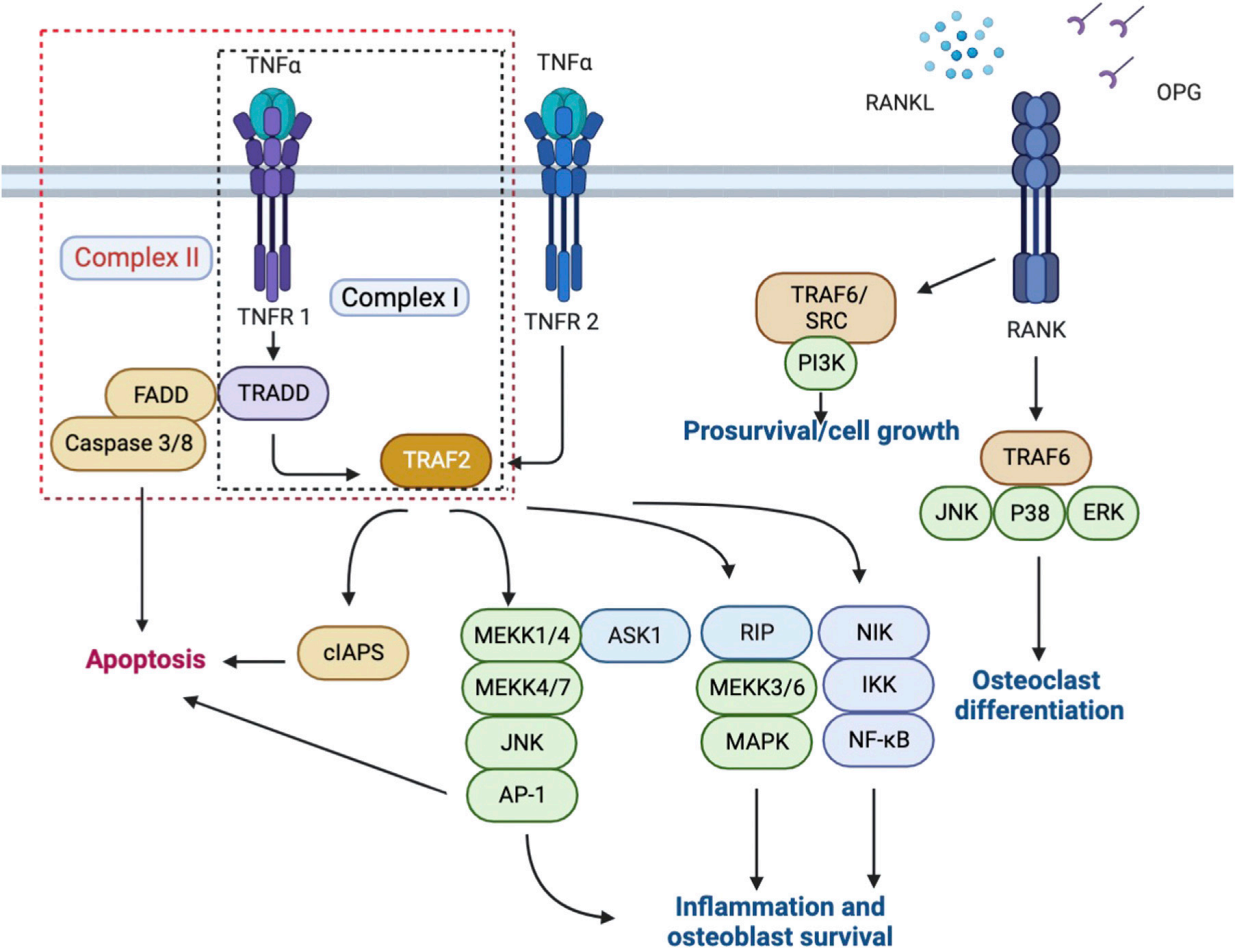
TNFR1, TNFR2 and RANK/RANKL/OPG intracellular signaling pathways.

TNFRSF is an important category of receptors for cytokines that provides crucial communication signals between various cell types during development and homeostasis, especially in the skin, bones, and lymphoid organs, and functions to maintain organ homeostasis and initiates tissue responses (Locksley et al., 2001). TNFRSF is further categorized into three groups according to different structures and functions, which are respectively characterized by death domains (DD) or death receptors, TNF receptor-associated factor (TRAF)-interacting receptors, and soluble or membrane-anchored TNFRSF receptors that act as decoy receptors of the death and TRAF-interacting receptors (Lang et al., 2016). Depending on different physiological processes, the consequences of signal transduction by TNF receptors include cell apoptosis, proliferation, or differentiation. During bone fracture healing, three members of TNFRSF are mainly involved, respectively TNFR1, TNFR2, and Osteoprotegerin (OPG), a decoy receptor for Receptor activator of nuclear factor-kappa-Β (RANK) that regulates the stimulation of RANK *via* competing for RANKL. (Figure 1).

### 1.2 TNFR1, TNFR2, and RANK

TNFα has long been identified as one of the most potent pro-inflammatory cytokines and a major driver of cell apoptosis and necroptosis *in vitro*. The anti-inflammatory effect of TNFR agonists and antagonists in the treatment of rheumatic diseases has been associated with their effect on blocking TNF from binding to TNFR1 and TNFR2.

TNFR1, also referred to as tumor necrosis factor receptor superfamily member 1A (TNFRSF1A), exerts its functions as a ubiquitous member receptor for TNFα. In bone, TNFR1 is expressed by both osteoblasts and osteoclasts (Bu et al., 2003). Once bound to TNFα, TNFR1 activates the mitogen-activated protein kinase (MAPKs) and canonical NF-kB pathways, and collectively led to the upregulation of pro-inflammatory genes transcription and results in inflammation. On the other hand, upon binding with TNFα, TNFR1 could also exert a cytotoxic effect *via* sequential assembly of a membrane-bound primary signaling complex (Complex I) that activates the formation of a secondary cytoplasmic complex (Complex II) and leads to cell death (Hsu et al., 1996; Micheau and Tschopp, 2003). However, it has now been increasingly clear that only when certain cell death checkpoints are activated would TNFR1 activation result in apoptosis or necroptosis, and in most cell types, TNFR1 activation instead triggers a pro-survival response. (Ting and Bertrand, 2016). This is achieved *via* the adaptor molecule TNFR1-associated death domain protein (TRADD), through which different signaling complexes could trigger either cell death or cell survival depending on the cellular context. (Brenner et al., 2015). TNFR2, or TNFRSF1B, is widely expressed by the immune, neuronal and epithelial cells, and has a high affinity for the membrane-bound form of TNFα, which leads to activation of the NF-kB and MAPKs pathways. (Al-Lamki and Mayadas, 2015; Mancusi et al., 2019). Apart from TNFR1, TNFR2 is the only membrane receptor that could bind to TNFα and exert signaling functions. Differences between TNFR1 and TNFR2 majorly lie in their intracellular structures; for instance, compared to TNFR2, TNFR1 contains an intracellular death domain (DD), which allows interaction between TNFR1 and DD-containing protein and evokes not only pro-inflammatory responses but also cytotoxic signaling. (Medler and Wajant, 2019). While TNFR2 plays a protective role upon interaction with TNF Receptor Associated Factor 2 (TRAF2) in many vital organs, the role of TNFR2 in the bone fracture healing process is more complicated and remains to be summarized. The polymorphisms in TNFRSF1B are often observed in patients suffering from rheumatic diseases, which seem to result in altered binding kinetics between TNFα and TNFR2 and eventually inhibition of the downstream NF-kB pathways, suggesting a protective role of TNFR2 signaling. (Yang et al., 2018).

RANK (TNFRSF11), RANKL, and osteoprotegerin (OPG) consist the signaling axis RANK/RANKL/OPG pathway, which plays a pivotal role in bone turnover in the context of inflammation. (Soos et al., 2022). Once activated by pro-inflammatory cytokines including TNF and interleukin, the RANKL-dependent pathways subsequently induce osteoclastogenesis, osteoclast differentiation, and osteoclast activation. (Schett and Gravallese, 2012; van den Berg and Miossec, 2009). In particular, upon binding with TNF, the expression level of RANKL by osteoblasts, B cells, and T cells is increased, which indirectly enhances osteoclastogenesis. (Lam et al., 2000; Schett and Gravallese, 2012). RANK/RANKL/OPG pathway has also been identified as an apoptosis regulator that maintains bone mass by regulating chondroclast differentiation, a crucial step during endochondral ossification. (Vu et al., 1998).

### 1.3 Current application of TNFRSF agonists and antagonists

Identified as inflammatory mediators, a growing body of evidence suggests that TNFRSF has the potential to be integrated into the new generation of biotherapy for the treatment of immune disease. (Croft et al., 2012). Currently, several anti-TNF drugs have been approved for clinical use to counteract the pro-inflammatory effect of TNF, which include infliximab (Remicade), adalimumab (Humira), certolizumab pegol (Cimzia), golimumab (Simponi), and etanercept (Enbrel). (Monaco et al., 2015). While these medications prove effective in the treatment of immune disorders including rheumatoid arthritis (RA) and inflammatory bowel disease (IBD), the side effects including opportunistic infections remains a problem for clinicians.

At the same time, the understanding of bone healing and reconstruction has advanced, and researchers nowadays hold the opinion that bone regeneration and reconstruction following a fracture are tightly regulated by both pro-inflammatory and anti-inflammatory cytokines, for example, the recruitment and activation of mesenchymal stem cells (MSCs) at the site of fracture to exert an anabolic effect on bone repair. (Liu et al., 2018). The future prospects of fracture treatment may, like many immune disorders, lie in local application of agonists and antagonists of TNFRSF at the site of fracture. (Table 1).

**TABLE 1:** Current medication options interfering with the TNFRSF signaling pathways. Abbreviation: CD - Crohn’s Disease; UC—Ulcerative Colitis; AS - Ankylosing Spondylitis; RA—Rheumatoid Arthritis; JIA - Juvenile Idiopathic Arthritis; PsA - Psoriatic Arthritis; HS - Hidradenitis Suppurativa; PsO - Plaque Psoriasis; URT—upper respiratory tract; (Information on Tumor Necrosis Factor, 2015; Medication Guide Prolia, 2022).

Drug	Route	Mechanism	Indications	Contraindications	Adverse
					Effects
Infliximab	Intravenous	Monoclonal antibody	CD; UC;	Hypersensitivity	Infections;
(Remicade)	Injection	Targeting	AS		Headache;
		TNFα			Abdominal
					pain
Adalimumab	Subcutaneous	Monoclonal antibody	RA; JIA;	None	Infections;
(Humira)	Injection	Targeting	PsA; AS;		Headache;
		TNFα	CD; UC;		Rash
			HS		
Certolizumab	Subcutaneous	TNF blocker	CD; RA;	Hypersensitivity	URT infections;
Pegol	Injection		PsA; AS;		Rash;
(Cimzia)			PsO		Urinary tract
					infections
Golimumab	Subcutaneous	TNF blocker	RA; PsA;	None	URT infections;
(Simponi)	injection		AS; UC		
Nasopharyngitis	Subcutaneous	TNF blocker	RA; JIA;	Sepsis	Infections;
Etanercept	Injection		PsA; AS;		Injection site
(Enbrel)			PsO		Reactions
Denosumab	Subcutaneous	RANKL	Conditions	Pregnancy;	Back pain;
(Prolia)	Injection	Inhibitor	that could	Hypocalcemia;	Arthralgia;
			reduce bone	Hypersensitivity	Musculoskeletal
			Mass		Pain

### 1.4 Bone fracture: burden, current treatment options, and future perspectives

Bone fracture is becoming a significant public health burden worldwide. In 2019, the total incidents of fracture reached 178 million in total, and the years lived with disability (YLDs) reached 25.8 million. (Collaborators, 2021). As an essential component of the musculoskeletal system, bone provides shape and support for the body and is subject to constant remodeling processes to maintain structural integrity and microarchitecture throughout life, and once fractured, these functions tend to be comprised, the quality of live lowered, and worse even, be lethal. (David and Schett, 2010). Promoting bone fracture healing in both time and quality is, therefore, an important measure to improve living standards.

Bone fracture healing is a multi-stage process that majorly involves two types of cells: osteoblasts and osteoclasts. Fracture healing can be divided into four stages, which are hematoma formation, intramembranous ossification, chondrogenesis, and endochondral ossification, each characterized by specific cellular events and extracellular matrix formation. (Bolander, 1992; Karnes et al., 2015). The healed bone will then undergo remodeling to improve the biological and physiological functions.

Current treatment methods for bone fracture vary depending on the sites and types. These methods generally include conservative treatment characterized by traction and external fixation, where the fracture is less severe and cause no blood flow disorder and nerve injuries, and surgical treatment followed by internal fixation, where circumstances are worse and influence the vessels and nerves. All methods, however, require several weeks to months for bone regeneration and functional rehabilitation to improve prognosis. The fixation process could be agonizing for patients, and clinicians are eager to find a new adjuvant therapy to facilitate fracture healing and lessen the timespan. As observed in the past, fracture healing is tightly regulated and the regulators including TNFRSF could be assimilated into the treatment.

While many studies have investigated the process of bone fracture healing, the involvement of signaling *via* TNFRSF in this process remains to be summarized. Therefore in this review, we seek to highlight processes where novel therapeutic interventions in TNFRSF signaling to enhance bone fracture healing are possible.

## 2 TNFR1, TNFR2 and RANK/RANKL/osteoprotegerin in bone fracture healing

### 2.1 TNFR1 signaling, osteoclastogenesis and fracture healing

To further discuss the roles TNFR1 plays in fracture healing, it is essential to understand two main stages of fracture healing, intramembranous and endochondral ossification. Intramembranous ossification occurs adjacent to the fracture site, where osteoblasts synthesize new bone tissue without cartilaginous intermediates. Endochondral ossification, by contrast, is the process of calcification in previously formed cartilages and continues until all cartilages are replaced by bone. While these processes are well-understood, many groups seek to study the roles TNFRSF plays in fracture healing on this basis. Lukić et al. (2005) found that while TNFR1 signaling does not affect intramembranous ossification, it is involved in endochondral ossification in adult mice. They then concluded that TNFR1 functions as a negative regulator during new tissue formation in endochondral ossification but not intramembranous ossification. (Lukic et al., 2005). In comparison, Gerstenfeld et al, (2001); Gerstenfeld et al., 2003)demonstrated that in TNFR1/2 double knockout mice, intramembranous ossification is also impaired, which indicates that TNFR2 could potentially play an imperative role in intramembranous ossification. To investigate whether TNFR1 regulates the formation and survival of osteoclasts and osteoblasts, Hiroki et al. (2010) used lipopolysaccharides (LPS) to induce bone loss in wild-type and TNFR1 knockout mice. (Ochi et al., 2010). The research group concluded that TNFR1 is indispensable in osteoclastogenesis and that TNFR1 is necessary to induce osteoclast precursors. More importantly, it is the first study to demonstrate that TNFR1 has an anti-apoptotic effect in LPS-induced inflammation *in vivo*. Furthermore, the study administrated osteoprotegerin (OPG) in mice with LPS-induced bone loss and determined that RANK/RANKL/OPG signaling affects osteoclast differentiation rather than the osteoclast precursor population.

In terms of disease management, although elevated TNFα levels have been identified in patients suffering from autoimmune and degenerative diseases, including Rheumatoid Arthritis (RA), and Irritable Bowel Disease, it is essential for maintaining homeostasis and fighting infections. While anti-TNF therapeutics have succeeded in the treatment of these autoimmune diseases, the severe side effects, including opportunistic infections, have limited further clinical application. (Monaco et al., 2015). To avoid side-effects caused by TNFα inhibition, Kontermann et al, (2008) developed a humanized TNFR1-specific antagonist designated as IZI-06.1 (Atrosab), an IgG1 molecule derived from mice immunized with human TNFR1. Concurrently as the molecule act as a potent TNFR1 antagonist, TNFR2 and other TNF-mediated immunity showed no significant signs of inhibition. In 2019, following this study, Richter et al, (2019) generated a monovalent anti-TNFR1 antibody fragment (Fab 13.7) and fused the variable heavy and light chains to the N-termini of the newly-formed heterodimerizing Fc chains. The constructed protein Atrosimab displays improved pharmacokinetic properties in the presence of anti-human IgG antibodies, retains strong binding to TNFR1, and shows a potent inhibition effect. The development of Atrosab and Atrosimab sheds light on the possibility that TNFR1 antagonists targeted to shorten the inflammation process and enhance new tissue formation during fracture healing be applied clinically without the risk of causing decreased immunity and opportunistic infections.

**FIGURE 2:**
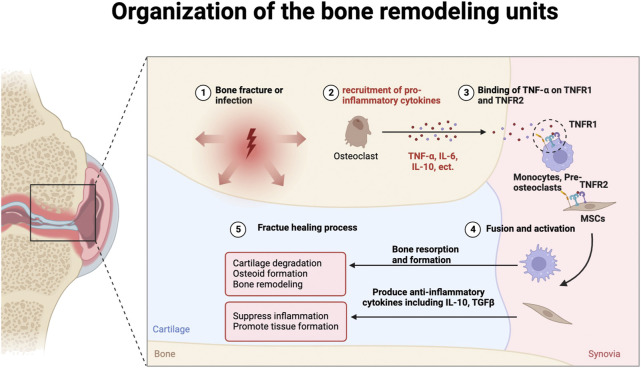
Organization of the bone remodeling units. MSCs: Mesenchymal stromal/stem cells.

To sum up, TNFR1 plays a pivotal role in osteoclastogenesis, functioning as a negative regulator in new tissue formation in bone fracture healing. TNFR1 antagonists, including Atrosimab and Atrosab, have seen tremendous advances in affinity to TNFR1 and potency as antagonists. The future perspective regarding bone fracture healing could assimilate the current understanding of TNFR1 signaling pathways and test the safety of these medications in phase 3 clinical trials while exploring other possible methods to deter TNFR1 signaling pathways and achieve faster recovery of bone fracture (Figure 2).

### 2.2 TNFR2, osteoclastogenesis, and osteogenic differentiation

TNFR2 expression, in comparison with the ubiquitous expression of TNFR1, is primarily on immune cells. (Medler and Wajant, 2019). Although signaling *via* TNFR1 and outcomes are well characterized, much less is known about TNFR2. (Sabio and Davis, 2014). Nagano et al. (2011) designed a study where they used subcutaneous injections of TNFα onto calvariae to induce bone resorption in both WT and TNFR2-deficient mice and observed the magnitude of bone resorption lacunae in each group to determine whether TNFR2 plays a protective role in the bone resorption process. (Nagano et al., 2011). The result showed that compared to mTNFα, hTNFα/Cholesterol-bearing pullulan (CHP) nanogel could significantly reduce bone mineral density (BMD), indicating that signaling *via* TNFR1 is essential for bone resorption induction and that signaling *via* both TNFR1 and TNFR2 could not induce bone resorption lacunae. To further rule out the possibility that TNFR2 plays no role in bone resorption, the research group used TNFR2-deficient mice and found that both hTNFα/CHP and mTNFα/CHP could significantly reduce BMD compared to the control group. These results, in summary, suggest that TNFR2 could have a suppressive effect against TNFR1-induced osteoclastogenesis in mice, which is the primary cause of bone resorption in chronic inflammatory diseases, and that TNFR2 agonists could be a potential therapeutic method in the treatment of bone fractures.

**FIGURE 3:**
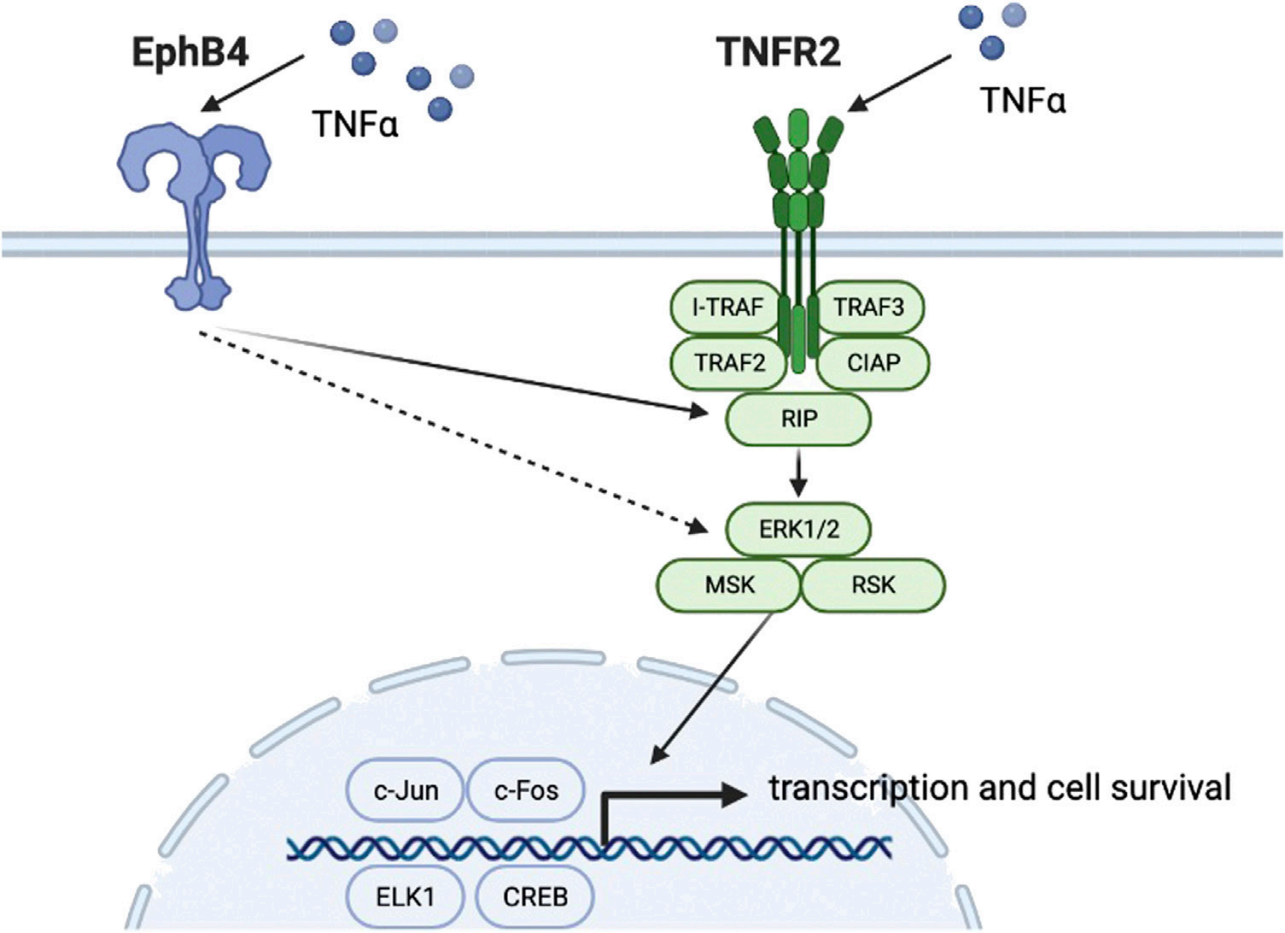
Schematic diagram of the EphB4, TNFR2 and ERK/MAPK signaling pathways.

With progressing studies into the effect of TNFR2 on osteoclastogenesis, other research groups seek to find whether the TNFR2 signaling pathway has a role in osteogenic differentiation. In 2020, Y Zhang et al. led a study on EphB4/TNFR2/ERK/MAPK signaling. (Zhang et al., 2020). Previous studies showed that low concentrations of TNFα and TNFR2 could upregulate EphB4 expression and promote osteogenic differentiation of osteoblast precursor cells. However, the roles of TNFR2 signaling and EphB4 in the osteogenic differentiation process and whether there is any crosstalk between these signaling pathways remain elucidated. Previous studies showed that TNFα could significantly enhance EphB4 expression. (Wang et al., 2017). Zhang et al, (2020) used lentivirus-mediated shRNA to knockdown TNFR2 expression level in MC3T3-E1 osteoblasts, which showed an approximately 70% reduction in TNFR2 expression compared with the control group. RUNX 2 and bone sialoprotein (BSP) were selected as indicators downstream of TNFR2 in osteoblast differentiation. The research group first observed that when treated with TNFα, RUNX2 and BSP levels in TNFR2 knockdown osteoblasts were significantly lower than that of the control group. In contrast, the expression level of EphB4 showed no significant difference between TNFR2 knockdown osteoblasts and the control group, indicating that TNFR2 activation does not affect TNFα-induced EphB4 upregulation (Figure 3). On the other hand, when EphB4 forward signaling was suppressed, TNFα-induced TNFR2 expression was lowered, which indicates that TNFα-enhanced EphB4 expression could upregulate TNFR2 expression. In this study, p38, *p*-p38, ERK1/2, *p*-ERK1/2, JNK1+2 + 3, and *p*-JNK1+2 + 3 levels were monitored under the aforementioned conditions and the result showed that in TNFR2 knockdown osteoblasts, the *p*ERK1/2 level was significantly decreased. Similarly, when EphB4 forward signaling was inhibited, *p*ERK1/2 level was also significantly down-regulated. When MC3T3 osteoblasts were pretreated with ERK inhibitor U0216, the levels of RUNX2 and BSP showed a significant decrease. It could be concluded that EphB4, TNFR2, and ERK/MAPK signaling pathways comprises a signaling axis that partly mediates the osteogenic differentiation induced by TNFα.

Efforts to target TNFR2 and achieve therapeutic effects in degenerative diseases involving TNFR2 activation, including RA and Alzheimer’s disease have been made. (Fischer et al., 2020). In 2011, R Fischer et al, (2011) constructed a soluble, human TNFR2 agonist *via* genetic fusion of the trimerization domain of tenascin C to a TNFR2-selective single-chain TNF molecule designated as TNC-scTNF. TNC-scTNF could bind to TNFR2 and induce clustering of TNFR2 on a cellular level. Later in 2014, R Fischer et al, (2014) managed to apply this method to generate a mouse TNFR2-selective mouse TNC-scTNF and incorporated D221N and A223R gene in mouse TNF to improve specificity. Furthermore, Fisher et al. (2017) applied tetramerization on the agonist and obtain improved crosslinking activity. (Fischer et al., 2017).

In summary, the TNFR2 signaling pathway shows a protective effect in bone resorption *via* suppression of TNFR1-induced osteoclastogenesis, and TNFR2 is possibly a part of a complex signaling axis that mediates osteoblast differentiation. Studies have shown that TNFR2 agonism has therapeutic potential in treating degenerative diseases. Therefore, the potential therapeutic value of TNFR2-targeting agents should not be overlooked.

### 2.3 RANK, RANKL, and OPG in bone modeling and remodeling

Chondroclasts, as another important component in fracture healing, are less well-understood than their counterparts, such as osteoclasts and osteoblasts. Ota et al. (2009) led research using wild-type and OPG double-knockout tibial fracture mice model to estimate the effect of OPG on fracture healing. The results showed that in OPG double-knockout mice, fracture healing is accelerated in that both the turnover rate of cartilaginous callus and the chondroclasts at the chondroosseous junction are increased and led to the conclusion that OPG deficiency could clearly promote chondrocyte-dependent chondroclastogenesis, accelerate the cartilage replacement by bone tissue and promote fracture healing. (Ota et al., 2009). These findings provide insights for accelerating fracture healing *via* applying OPG agonists or competitive inhibitors for RANK adjacent to the fracture site and promoting cartilage replacement.

**FIGURE 4:**
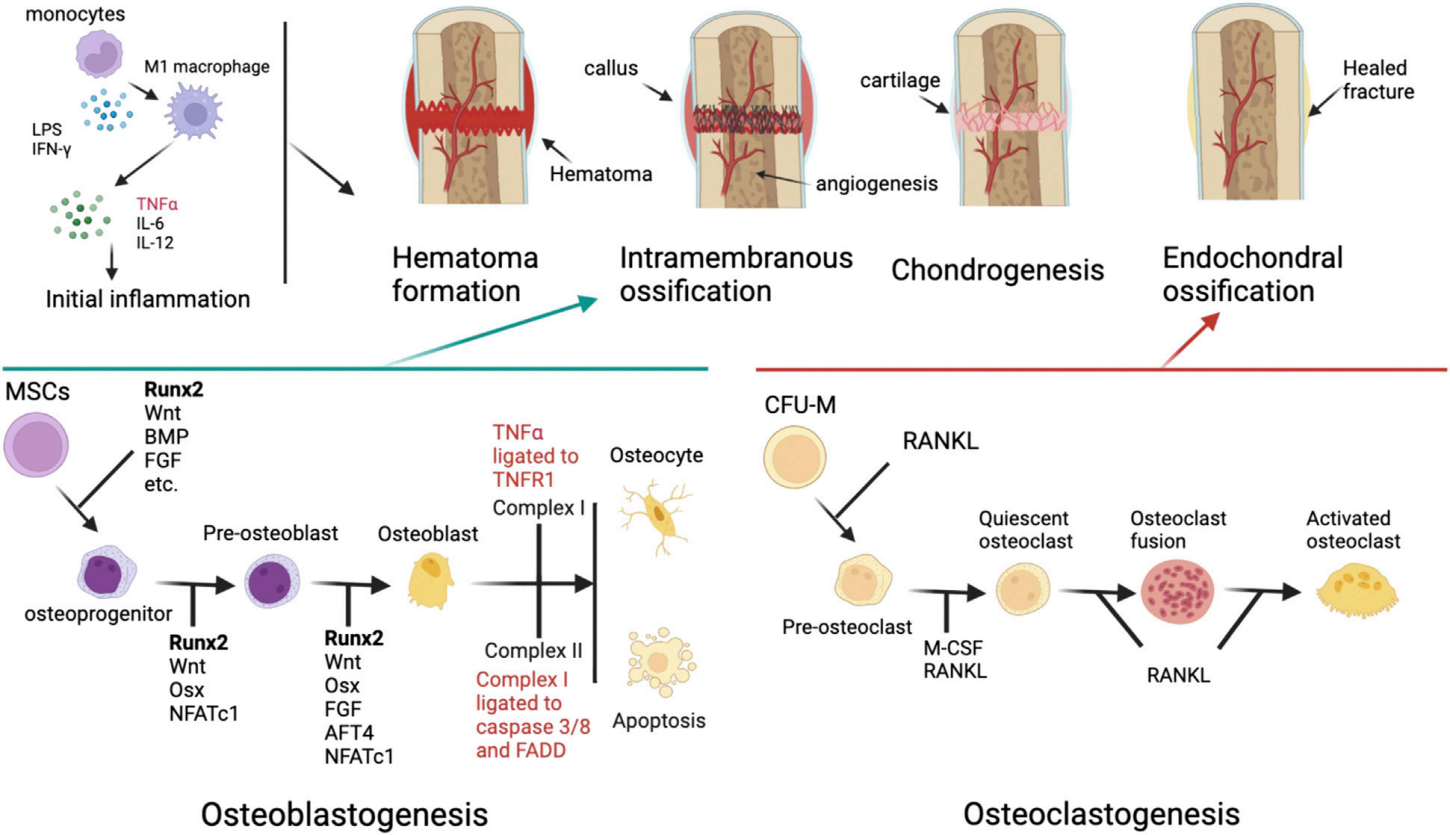
Interaction of exercise and RANKL/RANK/OPG biomolecular pathway.

One major component of bone fracture healing, besides the classic four-stage histological progression, is bone remodeling, a continuous process that guarantees the dynamic balance between bone formation and bone resorption. (Takayanagi, 2007). The bone mass can differ enormously depending on different ages (Santos et al., 2017), hormone levels, and weight-bearing levels (Figure 4). The RANK/RANKL/OPG pathway is the pivotal regulator of this process, as this signaling pathway plays a vital role in regulating osteoblasts and osteoclasts. (Boyce and Xing, 2007). For patients suffering from bone fracture, one of the most effective methods following the treatment is to seek help from physical medicine or rehabilitation since studies have shown that exercise could facilitate bone modeling and remodeling. (Qi et al., 2016).

Although the role of the RANK/RANKL/OPG signaling pathway in bone modeling and remodeling is researched by many groups, the scope of this matter remained at an individual level. To better understand whether the RANL/RANKL/OPG signaling pathway is associated with the risk of fracture in the general population, P. Tharabenjasin et al, (2022) conducted a systematic review in 2021 where they searched 13 articles available concerning OPG and fracture and examined four polymorphisms of genes encoding OPG. The result showed that OPG gene polymorphisms could reduce the risk of osteoporotic fracture in the senior population, especially in postmenopausal women over 60 years of age. This result shed light on the possible application of OPG gene sequencing in the population as early detection of possible osteoporosis or other diseases that could cause predisposition to bone fracture, followed by an early intervention to avoid such situations. The statistical result indicates the potential future application of RANK/RANKL/OPG is in the early detection and intervention of diseases or conditions that could cause bone fracture.

In fact, the attempt to target RANKL *via* artificial monoclonal antibody is not unprecedented. Raje et al. (2018) conducted a double-blinded, randomized phase-3 clinical study in adult patients suffering from newly diagnosed multiple myeloma (MM) with at least one lytic bone lesion. The research team selected denosumab, a monoclonal antibody targeting RANKL, to compare the safety and efficacy in preventing skeletal-related events caused by MM compared with zoledronic acid, an intravenous biphosphate. The study continued from May 2012 to March 2016, and a total of 1718 patients were included. At the endpoint, denosumab achieved non-inferiority compared with zoledronic acid, suggesting that denosumab could be an additional therapeutic option for MM patients suffering from skeletal implications. (Raje et al., 2018).

## 3 Conclusion

**FIGURE 5:**
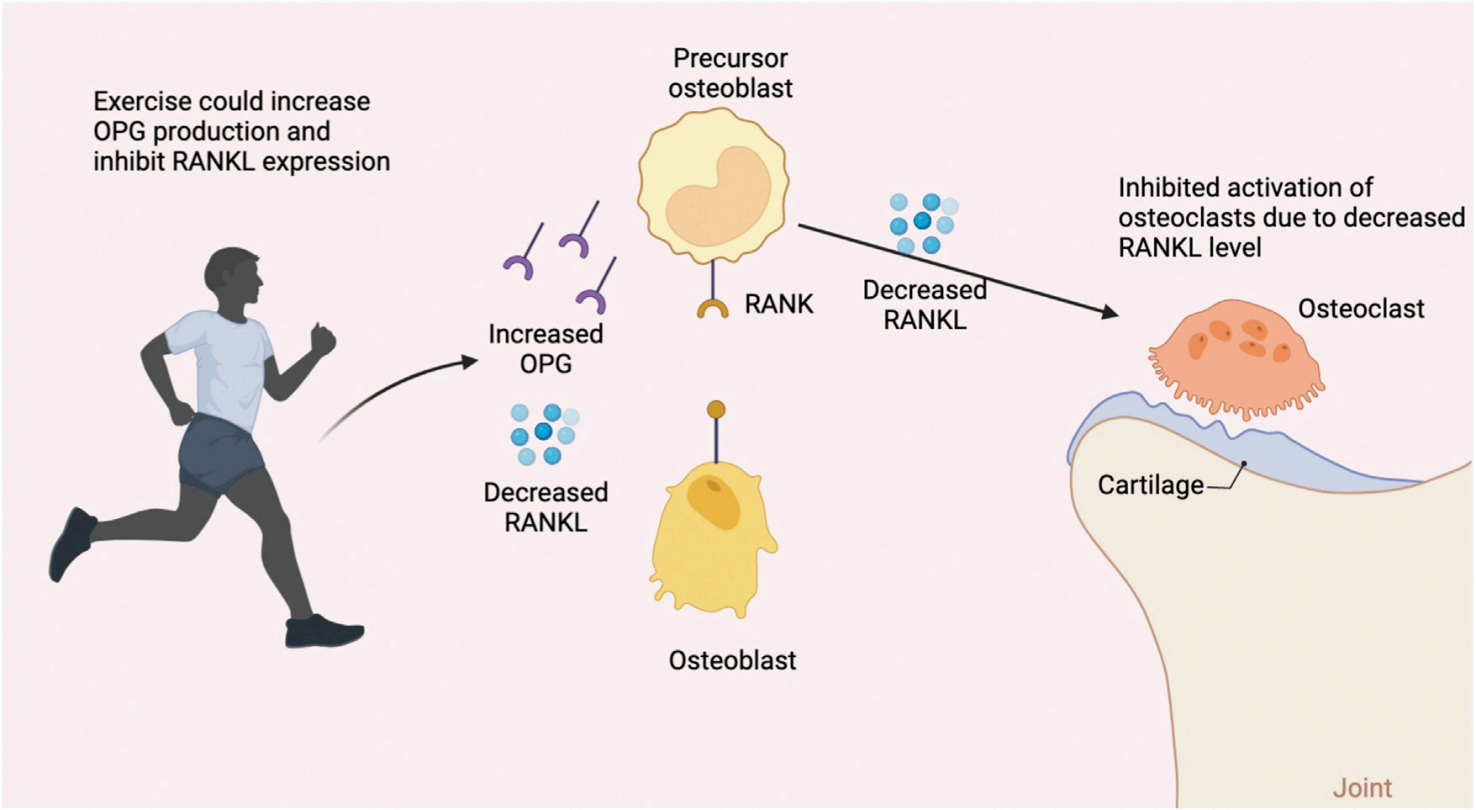
General overview of the four-stage fracture healing process and the respective signaling pathways and cytokines involved in initial inflammation, osteoblastogenesis, and osteoclastogenesis. As mentioned in 2.2, the Runx2 level is potentially associated with TNFR2 signaling. (Zhang et al., 2020) MSCs: mesenchymal stem cells; CFU-M: Colony forming unit M, namely monoblast. M-CSF: macrophage colony-stimulating factor.

In the musculoskeletal system, TNFRSF members exert different functions and form a complex signaling network. TNFR1 has a crucial role in osteoblastogenesis and osteoclastogenesis, where it could either facilitate osteoblast differentiation and osteocyte survival or result in osteoblast apoptosis, while TNFR1 is also indispensable for osteoclast differentiation and activation. While the underlying mechanism that modulates osteoblastogenesis remains unclear, TNFRSF-targeted antagonists or other agents could selectively bind to TNFR1 and mediate osteoclasts formation, and therefore, facilitate endochondral ossification. This also applies to TNFR2-targeted agents, since TNFR2 has been discovered as a possible member of the EphB4 and ERK/MAPK signaling axis and that TNFR2 plays a vital role in osteogenic process and osteoclastogenesis. Similarly, for other members of TNFRSF, the therapeutic potential in bone fractures and other musculoskeletal diseases or events should not be ignored. In the RANK/RANKL/OPG pathway, monoclonal antibodies including denosumab have already emerged, confirming the therapeutic value of these targets (Figure 5.).

For future perspectives on TNFRSF-targeted therapeutic regimens, one possible form of treatment is the implantation of hydrogel or other matrix loaded with medication at the fracture site, which could eliminate disseminated adverse effects and increase drug concentration to achieve a better therapeutic effect. In conclusion, TNFRSF has excellent therapeutic potential in treating and preventing skeletal-related diseases, including bone fractures. Indeed, the future application of such therapy may emerge in various forms, and this field remains in need of further investigation and experimentation.

## Data Availability

The original contributions presented in the study are included in the article/supplementary material, further inquiries can be directed to the corresponding author.
